# Advances in neuroRehabilitation of TREM2-related dementia: A case report on a novel multimodal approach using virtual reality

**DOI:** 10.1097/MD.0000000000029470

**Published:** 2022-05-27

**Authors:** Rosaria De Luca, Angela Marra, Patrizia Pollicino, Marella Buda, Maria Mucari, Mirjam Bonanno, William Torregrossa, Angelo Caminiti, Carmela Rifici, Rocco Salvatore Calabrò

**Affiliations:** IRCCS Centro Neurolesi “Bonino Pulejo”, Messina, Italy.

**Keywords:** advanced neurorehabilitation, behavioral and cognitive deficits, fronto temporal dementia, TREM2 mutations, virtual reality rehabilitation system-evo

## Abstract

**Rationable:**

The aim of this study is to investigate the effects of an advanced neuroRehabilitation protocol using virtual reality in the treatment of a patient with fronto- temporal dementia due to TREM2 mutation.

**Patient's concern:**

A 41-year-old caucasian male, affected by Nasu-Hakola Disease (NHD), presented a 1-year history of change in behavioral and cognitive functioning, before our observation. The onset of the disease was characterized by severe pain in the lower limbs and knees with limitations in the performance of daily life activities.

**Diagnosis:**

Motor and cognitive deficits in NHD.

**Interventions:**

As the patient was in a chronic phase, to manage his cognitive and motor status, we decided to treat him by using a specific rehabilitation protocol, including 2 different types of training: conventional cognitive and motor treatment and a combined advanced approach using the virtual reality rehabilitation system (VRRS). The two protocols were separated by 4 weeks of rest, to avoid/reduce a cumulative effect. The patient's cognitive and motor profile was assessed three times: that is before (at T0) and after (at T1) the conventional training as well as at the beginning (T2) and at the end of the combined experimental approach (T3).

**Outcomes:**

After the combined therapeutic approach with the virtual reality rehabilitation system, we observed a significant reduction in anxiety, apathy, indifference and depressive symptoms with a more evident motor improvement involving the head and the trunk control.

**Lessons:**

Virtual reality can be considered a promising tool for the motor and cognitive rehabilitation of rare neurodegenerative disorders, including patients with NHD.

## 1 Introduction

Nasu-Hakola disease (NHD) is a rare autosomal recessive disease characterized by progressive presenile dementia (similar to the behavioral and cognitive variant of fronto-temporal dementia – fronto-temporal dementia [FTD]) associated with the formation of painful multifocal bone cysts and fractures (both evident by the third decade). NHD is caused by genetic mutations in the TREM2, a transmembrane receptor of microglia, which plays an important role in neuroinflammation.^[1–3]^ The majority of patients have been reported in Finland and Japan,^[4]^ but there are less than 200 cases in the literature,^[5]^ FTD has a slow and insidious onset and it is typically diagnosed during middle age. Most affected individuals are aged 45 to 65 years; in this age-group, FTD is estimated to affect 10 to 15 individuals per 100,000 of the population.^[6–8]^ Clinical manifestation of NHD may present with involvement of the central nervous system (CNS) in the absence of characteristic skeletal symptoms, with cognitive decline and behavioral changes.^[9,13]^

A few cases with homozygous mutations in *TREM2* have the atypical phenotypes resembling bvFTD^[10–13]^ In most Nasu-Hakola patients, the clinical course of the disease can be divided into 4 stages: (1) the latent stage with normal early development; (2) the osseous stage beginning at the third decade of life, characterized by pain and swelling in ankles and feet followed by frequent bone fractures; (3) the early neuropsychiatric stage occurring at the fourth decade of life, presenting with a frontal lobe syndrome including euphoria and social disinhibition; and (4) the late neuropsychiatric stage, characterized by profound dementia, loss of mobility, and death usually by age of 50 years.^[14]^ Nowadays, the current literature addresses the disease exclusively from a neurobiological^[15]^ and genetic^[16]^ view or, in limitate cases, neuropsychological assessment has been reported.^[17,18]^ However, no case has to date dealt with both cognitive and physical neurorehabilitation, because of both the rarity of the disease and its inexorable progression.

The aim of this study is to investigate the beneficial effects of an advanced neurorehabilitation multimodal approach, including virtual reality, in a patient with fronto – temporal dementia due to TREM2 mutation.

## 2 Case description

We report a 41-year-old caucasian (Italian) male patient, who came to our observation after a 1-year history of change in behavioral and cognitive functioning. Some years before, he was diagnosed with NHD, caused by a genetic mutation in the TREM2.

His parents were both clinically healthy, but they were carriers of the disease. His brother had the same TREM2 gene mutation, whose onset, unlike his brother, was characterized by cognitive dysfunctions, including amnesia, memory alterations and more evident behavioral problems, with a gradual deterioration of the clinical picture until his death two years ago. Instead, the affected sister had only an increase in muscular-articular pains. Moreover, three of his first cousins as well as her eldest daughter also had febrile seizures in childhood, like the patient had.

At the disease onset (about 10 years ago) the patient began to present with severe pain in the lower limbs and knees with disabling slow, but progressive, limitations in the autonomy of daily life activities. Since 2017 the clinical picture has worsened, as the wife reported frequent forgetfulness including appointments, conversations, with important difficulties in the organization of everyday life. He also presented an increasing apathy and reduced empathy towards friends and family. The patient progressively showed non-contextual behavior with a tendency to disinhibition (such as actions and gestures that did not adhere to reality with inappropriate statements). In the last period, further symptoms, such as evident hand tremors and urgency to urinate, especially in the morning, appeared. Finally, his wife noticed that he had started to become more persistent in his behavior, developing a change in food preferences by eating more junk food. He also began to have mild difficulty finding words with frequent anomia.

The neurological examination revealed a postural tremor associated with a small step gait and mixed hypertonus. At the neuropsychological examination, severe spatial and temporal disorientation, deficits in visual-executive functions, particularly abstract thinking, planning and inhibition were found. There was also evidence of language dysfunction with nonfluent speech, anomia, and phonemic paraphasias; although comprehension and repetition are incostant and very slowed, with a reduced verbal initiative (verbal inertia). We have also observed a severe alteration of mood with a tendency to apathy.

Blood tests, including serum calcium, phosphate, and parathyroid hormone were normal. Electroencephalography was characterized by slow theta waves, poorly organized with irregular morphology in the parieto-temporal region of the right hemisphere.

Magnetic resonance imaging of the brain showed atrophy mainly located in the frontal, lateral temporal, and parietal cortices and also the pallidus nuclei with marked thinning of the bilateral sotto-cortical region and periventricular white matter lesions. Skeletal x-ray reveals severe radiolucency detected in the upper and lower limbs.

### 2.1 Procedures

As the patient was in a chronic phase, to manage his cognitive and motor status, we decided to treat him by using a specific rehabilitation protocol, including 2 different types of training. The patient was adequately informed about the study and offered his collaboration and written consent.

In the 1st phase, the patient received a conventional treatment, including conventional cognitive training (CCT) and standard physiotherapy (SPT). After the first phase, 2 weeks of latency have elapsed before the next phase, in which the patient was only treated with physiotherapy (to avoid a cumulative effect). Then, the patient was submitted to the experimental protocol (second phase; i.e. a combined therapeutic approach) by which cognitive and motor training was provided using the virtual reality rehabilitation system (VRRS, by Khymeia, Italy). However, the patient was provided with the same amount of treatment during both the training phases. In particular, in the first phase the patient received both SPT and CCT, 5 days a week for 12 weeks (for a total of 60 sessions each), whereas in the second phase the patient was submitted to 30 SPT sessions and 30 CCT sessions, in addition to 60 VRRS sessions (both cognitive and motor) (Table 1).

**Table 1:** TREM2 Neuro_Rehabilitation Program: session duration and type of treatment.

Rehabilitation program	Rehabilitative intervention	Session duration	Type of treatment
Step 13 mo(Sep–Nov 2021)*Conventional Neurorehabilitation*	CCT	6 weekly sessions of 60 min (72 total treatments)	ROT 15 min: 5 min Temporal Orientation5 min Spatial Orientation5 min Personal Orientation (Emotional/Autobiographical/Motivational Training)
			APT 20 min: 5 min Visual Research/Selective Attention5 min Alternating Attention5 min Divided Attention5 min Sustained attention
			Memory Training 10 min:5 min Visuo-spatial memory5 min Verbal Memory
			Executive Education Training 15 min10 min Verbal Fluency5 min Categorization
	S-PT	6 weekly sessions of 60 min (72 total treatments)	Physical Rehabilitation15 min manipulation on muscle tissues, stretching and mobilization in supine position10 min active assisted exercises20 min exercises for trunk control and balance in sitting position/core stability training in supine positionRespiratory Gymnastics10 min Mobilization of the diaphragm muscle5 min Breathing control training
Step 23 mo(Dec 2021–Feb 2022*)Combined Rehabilitative Approach*	CCT	3 weekly sessions of 60 min (36 total treatments)	ROT 15 minAPT 20 minMemory Training 10 minExecutive Education Training 15 min (the aforementioned program)
	Standard Physiotherapy (S- PT)	3 weekly sessions of 60 min (36 total treatments)	Physical Rehabilitation 35 minRespiratory Gymnastics 25 min (the aforementioned program)
	VRRS–Software and Tools dedicate for *Cognitive Module*	3 weekly sessions of 60 min (36 total treatments)	ROT 15 minAPT 20 minMemory Training 10 minExecutive Education Training 15 min (the aforementioned program)
	VRRS – Software and Tools dedicate for *Physical Module*	3 weekly sessions of 60 min (36 total treatments)	Physical Rehabilitation 45 minRespiratory Gymnastics 15 min (the aforementioned program)

APT = process training, CCT = conventional cognitive training, ROT = reality orientation therapy, S-PT = standard physiotherapy, VRRS = virtual reality rehabilitation system.

Written informed consent was obtained from the guardian of the patient for publication of this case report and accompanying images

### 2.2 Functional assessment and analysis

The psychometric battery included: the Mini Mental State Examination^[19]^ to evaluate the global cognitive status; the Brief Psychiatric Rating Scale^[20]^ to assess the presence and entity of psychiatric symptoms; the Frontal Assessment Battery^[21]^ to investigated the verbal and not executive functions; the Functional Communication Scale^[22]^ to assess the communicative skills, the Clock Drawing test to explore the visuo-spatial research and orientation,^[23]^ and Hamilton Rating Scale for Depression to investigate the level of depression symptoms (Table 1).^[24]^

The motor and functional tests/scales included: the Disability Rating Scale (after discussion with the rehabilitation team) to evaluate functional assessment measure^[25]^; the Functional Independence Measure,^[26]^ an instrument that was developed as a measure of disability and Trunk Control Test ^[27]^ that scores motor impairment, trunk balance and coordination (Table 2).

**Table 2:** Patient's neuropsychological and motor assessment.

Test/Scale	Domains	Description
Mini Mental State Examination (MMSE)	Global Cognition	MMSE is a set of 30 questions that doctors and other healthcare professionals commonly use to check for cognitive impairment (problems with thinking, communication, understanding and memory). It includes tests of orientation, attention, memory, language and visual-spatial skills. The total score is between a minimum of 0 and a maximum of 30 points. A score equal to or less than 18 indicates a severe impairment of cognitive abilities; a score between 18 and 24 indicates moderate to mild impairment, a score of 25 is considered borderline, and a score of 26 to 30 indicates cognitive normality.
Brief Psychiatric Rating Scale (BPRS)	Psychiatric Symptoms	BPRS may use to measure psychiatric symptoms such as depression, anxiety, hallucinations and unusual behaviour. The scale is one of the oldest, most widely used scales to measure psychotic symptoms.The BPRS consists of 18 items measuring the following factors: anxiety, emotional withdrawal, conceptual disorganization, guilt feelings, tension, mannerisms and posturing, grandiosity, depressive moods, hostility, suspiciousness, hallucinatory behavior, motor hyperactivity, uncooperativeness, unusual thought content, blunted affect, somatic concern, excitement, and disorientation.
Frontal Assessment Battery (FAB)	Executive Functions	The FAB is a brief tool that can be used at the bedside or in a clinic setting to assist in discriminating between dementias with a frontal dysexecutive phenotype and Dementia of Alzheimer's Type (DAT). The FAB has validity in distinguishing Fronto-temporal type dementia from DAT in mildly demented patients (MMSE > 24). Total score is from a maximum of 18, higher scores indicating better performance.
Functional Communication Scale (FCS)	Communication Abilities	FCS is a specialist language questionnaire of verbal and non-verbal abilities to investigate global communication, which evaluates the language abilities (verbal and non-verbal communication skills); it is carried out by the speech therapist to investigate the different items: motivation, collaboration, understanding and language abilities; Response options range from 0 to 22.
CDT (Clock’drawing test)	Executive Function Visual-Spatial Processing	CDT is a non-verbal screening tool in which the patient is asked to draw a clock. It is used to quickly assess visuospatial and praxis abilities, and may determine the presence of both attention and executive dysfunctions; The CDT may be used in addition to other quick screening tests such as the Mini-Mental State Examination (MMSE), and the Functional Independence Measure (FIM). The patient is then asked to draw the hands on the clock to indicate “10 min past 11 o’clock.” Moreover, it also assesses long-term memory, auditory processing, motor programming, and frustration tolerance. The maximum score of the free drawing version is 15 (only this condition is not adjusted and corrected for age); the maximum score of the pre-drawn clock is 13, the maximum score of the designed clock by the examiner is 33.
Hamilton Rating Scale for Depression (HRS-D)	Depression symptoms	HRS-D is the most widely used clinician-administered depression assessment scale. A later 21-item version (HDRS21) included 4 items intended to subtype the depression, but which are sometimes, incorrectly, used to rate severity. Method for scoring varies by version. Not depressed: 0–7; Mild (subthreshold): 8–13; Moderate (mild): 14–8; Severe (moderate): 19–22; Very severe (severe): >23.
Disability Rating Scale (DRS)	Level of Disability	DRS is primarily used to assess impairment, disability, and handicap of an individual. An impairment rating is based on the Glasgow Outcome Scale, such as “Eye Opening,” “Communication Ability,” and “Motor Response.” Disability assesses the cognitive ability of the individual. Score from 0 – Normal status to 29 – Extreme Vegetative State (or possible death).
Functional Indipendence Measure (FIM)	Functional Status	The FIM is an ordinal scale composed of 18 items with seven levels ranging from 1 (total dependence) to 7 (total independence) designed to determine the level of disability of patients, as reflected by their need for assistance and/or aids during the execution of activities of daily living.The FIM can be subdivided into a 13-item motor subscale (motFIM) and a 5-item cognitive subscale (cognFIM). The ranges of scoring for the motor and cognitive subscales are 13 to 91 and 5 to 35, respectively. A good interrater reliability has been demonstrated both for the TCT and for the FIM.
Trunk Control Test (TCT)	Trunk Movement Patterns	The TCT examines four axial movements: rolling from a supine position to the weak side (T1) and to the strong side (T2), sitting up from a lying-down position (T3), and sitting in a balanced position on the edge of the bed with feet off the ground for 30 s (T4). The scoring is as follows: 0, unable to perform movement without assistance; 12, able to perform movement but in an abnormal manner; and 25, able to complete movement normally. The TCT score is the sum of the scores obtained on the four tests (range, 0–100). The examiner's score must relate solely to the performance during the test and not be based on referred data.

Both the neuropsychologist and physiotherapist, who administered the scales, were blinded to the patient's treatment (Table 3).

**Table 3:** Cognitive Rehabilitative program including the standard and the experimental (VRRS) one.

		*Conventional Training (CT)*Paper and pencil task - sensory stimulationFace to Face rehabilitative settingDirect interaction only between patient and her/his therapist3 levels of complexity for execution's time and the numbers of stimuli-target and distractores administered	*VRRS - Training (VRRS)*Pc-based task - Augmented sensory feedbackHuman - web setting interfaceVirtual interaction mediated by VRRS tool3 levels of difficulty for execution's time and the numbers of stimuli-target and distractores administered
*Cognitive Domain*	*Sub-items*	*CT-Task for specific domains*	*VRRS-Task for specific domains*
Orientation	Personal Orientation	To observe and select the emotional traditional pictures - personal setting - autobiographical photographs (about home, family, friends, pets, wife, mother, daughter etc ..); Voice recordings on smartphones, listening personal audio-video materials such as voice recordings family, friends, relatives, colleagues; music tracks - emotionally meaningful songs; main list of favorite movie scenes; videos of personal life scenes (marriage - birth of children …).	To observe and select the emotional virtual pictures - personal setting - autobiographical virtual photographs (about home, family, friends, pets, wife, mother, daughter etc ..);Using VRRS, integrated to the virtual system, listening to personal audio-video materials such as voice recordings of family, friends, relatives, colleagues; music tracks - emotionally meaningful songs; main list of favorite movie scenes; videos of personal life scenes (marriage - birth of children …).
	Spatial Orientation	To stimulate spatial orientation through the recall of places, itineraries and spatial locations, using systematic paper cards of Manual of Cognitive Training. Execution of virtual spatial orientation activities, spatial awareness activities: traditional puzzles (different scenes/pictures), 2D Blocks’ position (centre, right - left), rotation of objects, draw and paint, explore maps and explore shapes.	To stimulate orientation in the space, spatial sense, spatial perception, spatial skills, spatial reasoning activities through the recall of places, itineraries and spatial locations. Execution of virtual spatial orientation activities, spatial awareness activities: Virtual Puzzles (different scenes/pictures), Virtual Block's position (centre, right - left), Virtual rotation of objects, Virtual draw and paint, explore interactive maps and explore pc-shapes.
	Temporal Orientation	To stimulate temporal orientation through the recall of days, months, years, festivities, events of personal data, personal stories. The information is repeatedly transmitted in visual, verbal, written or auditory modality.	To stimulate temporal orientation throuht the recall of days, months, years, festivities, events of personal data, personal stories, using VVRS software dedicated. The information is repeatedly transmitted in pc-based visuo-verbal, written or auditory modality.
Attention Processes	Selective	To indicate and touch directly with his hand the selected/standard target -stimuli in relation to specific characteristics presented (color, image, animals, function…) neglecting the distractions, which consist in other pictures, different for number and complexity of criteria. Cognitive therapist showed the verbal commands to the patient, which combined the different selective images. The patient touches the standard target stimuli presented in a specific time, according to the therapist's verbal command.	To select and immediately recall feedback (audio and video) similar to various elements: colours, musical strings, geometric or not form, animals …obseved in the virtual enviroument. The patient touches the virtual target element in a specific time, this action causes a visual change with a specific audio feedback (positive reinforcement), using VVRS - interaction between the cognitive therapist and patient. Otherwise the element disappears (negative reinforcement).
	Alternating	To increase the attention alternating processes, the cognitive therapist organized specific activities, involving the mental flexibility for moving between tasks with different cognitive requirements, which use pencil-and-paper tasks (such as to make simple sequences of animals, fruits, objects - colors - pictures).	To increase the attention alternating processes, the cognitive therapist selected specific virtual activities, involving the mental flexibility for moving between tasks with different cognitive requirements, which use computer games/software dedicated (such as to make simple sequences of animals, fruit, objects - colors - pictures).
	Sustained	To stimulate sustained attention processes, the patient observed from 3 to 5 targets–stimuli for a variable and progressive time (10–15 min), with an attentional focus on traditional tasks.	To stimulate sustained attention processes, the patient observed from 3 to 5 targets–stimuli for a variable and progressive time (10–15 min), with an attentional focus on virtual tasks.
	Split	The therapist asks the patient to perform a double task such as selecting/associating the color to the shape and at the same time eliminating the different standard stimuli.	The therapist asks the patient to perform a double task such as selecting/associating the color to the shape and at the same time eliminating the different shapes/virtual stimuli.
Memory	Verbal	To work on recognition and remembrance traditional tasks with verbal material, reminiscence and validation therapy, mnemonic techniques and strategic skills.	To work on recognition and remembrance virtual tasks with verbal material, reminiscence and validation therapy, mnemonic techniques and strategic skills.
	Visuo-spatial	To work on recognition and remembrance traditional tasks with verbal material, reminiscence and validation therapy, mnemonic techniques and strategic skills.	To work on recognition and remembrance virtual tasks with verbal material, reminiscence and validation therapy, mnemonic techniques and strategic skills
Exsecutive Functions	Verbal Fluence Reasoning	The training of the executive function was reached by working on categorization (semantic and phonemic), planning, association and analogical reasoning, without the use of virtual tool.	The training of the executive function was reached by working on categorization (semantic and phonemic), planning, association and analogical reasoning, using pc-based approach.

We evaluated the patient's cognitive and motor profile in 2 separate phases, before and after the 2 different trainings (T0: baseline; T1: at the end of the traditional neurorehabilitation training; T2: at the beginning of the combined experimental approach; and T3: after the combined approach).

Reliable change index (RCI) was used to evaluate whether a change in an individual's score (i.e., between T0 and T1, and T2 and T3) was significant or not (based on how reliable the measure is). We used RCI to define if the change observed in the patient was clinically and practically significant, based on the amount of change that a patient had shown on a specific psychometric instrument between the different assessments. The RCI is a statistic used to determine if a change occurring in the score of an individual (or group) is statistically significant based on the test-retest reliability of the measurement.^[28]^ This provides information about the likelihood that a change in test scores “results from” true or reliable change or results from the case^[29]^ Given that it has been demonstrated that RCI could be useful in assessing changes in the neuropsychological field,^[30]^ we used this index also for motor functional outcomes.

### 2.3 Conventional rehabilitation

The conventional treatment consisted of CCT and SPT. Physiotherapy was carried out through manipulation of the muscle tissue, passive mobilization of the four limbs to ensure a better articular excursion and prevent muscle-tendon retractions, and stretching of the sternocleidomastoid and cervical paravertebral muscles in order to align the head. Then, active-assisted exercises were performed aimed at increasing muscle strength especially in the lower limbs (through isometric contractions of the gluteal and quadriceps muscles), as well as to improve head and trunk control by potentiating core stability muscles. In particular, to rebuild the control of the patient's trunk, the physiotherapist proposed exercises to shift the center of gravity in both antero-posterior and latero-lateral directions, thus training postural feedback and feedforward mechanisms. Finally, the patient, through the sitting position, experimented with manipulative and reaching proposals for the recovery of the motor skills of the upper limbs and for reactivating the eye-hand coordination. This rehabilitation program allowed the patient to achieve the sitting position, once he had acquired control of the head and trunk and had obtained active motility in the upper limbs.

CCT was based on a face-to-face approach between the therapist and the patient using paper and pencil tools. It was mainly focused on strengthening orientation with a specific cognitive program based on the reality orientation therapy; autobiographical memory, temporal and spatial orientation, and simple relationships and logic associations were also trained. To improve attention, we used the attention process training that includes task targeting for sustained, selective, split and alternating attention. The memory enhancement goal was achieved by working on recognition and remembrance tasks with verbal and nonverbal material, reminiscence and validation therapy, mnemonic techniques and strategic skills. The training of the executive function was reached by working on categorization, planning, association and analogical reasoning. CCT was focused to reduce depression symptoms, using a narrative and introspective training to support the patient (Table 3).

### 2.4 Experimental training using VRRS

**Figure 1:**
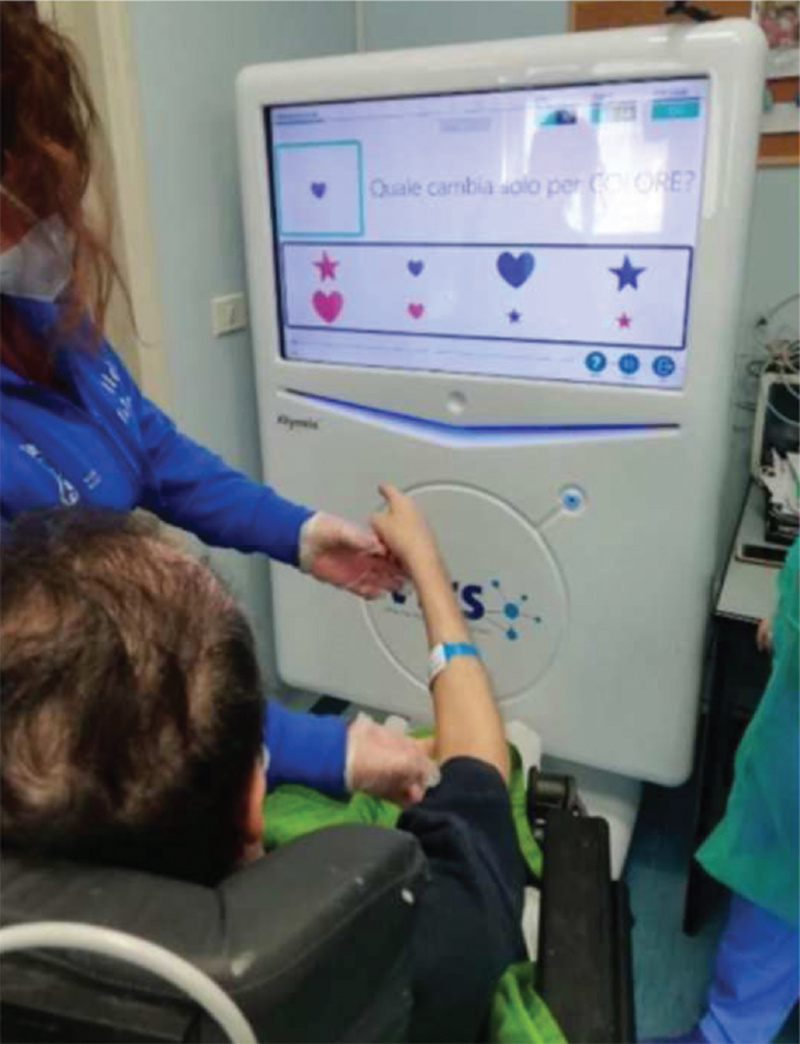
The patient is seated in a wheelchair in front of the VRRS station, supervised by the constant presence of the physiotherapist while carrying out manual eye coordination exercises, task-oriented pointing. In particular, we observe in this picture, that the patient selects the target stimulus, indicating, among some distractors, the correct response related to shape and color of the stimuli through the movement of the upper limb and the fine motility of the fingers. VRRS = virtual reality rehabilitation system.

The experimental protocol was provided by the VRRS, one of the most advanced comprehensive and clinically proven virtual reality (VR) systems for rehabilitation^[31–32]^ and telerehabilitation. Extremely easy to use, high customization capacity, complete automated reporting and functional telerehabilitation, are some of the guiding principles of continuous system development (Fig. 1). The VRRS, in fact, is conceived as a “central HUB” to which, via USB it is possible to connect a series of specialized peripherals, fully synchronized and integrated with the system. The VRRS can be used in many neurological disorders, thanks to the different modules for cognitive, language, postural, and motor rehabilitation.^[32]^ In this protocol, we used the cognitive and motor modules.

The VRRS cognitive module consists of a large set of activities for rehabilitation, with more than 50 exercises already available and many others under development. All activities are organized by cognitive function: memory, attention, language, spatial-temporal orientation, planning, reasoning and other executive function, calculation, and praxis. Cognitive exercises provided through the VRRS can be classified in 2 main categories. The 1st category includes 2D exercises where the patient interacts with objects and scenarios through the touch screen or through a particular magnetic tracking sensor coupled with a squeezable object, thus emulating mouse-like interaction capabilities. The 2nd category consists of 3D exercises, where the patients interact with 3D on immersive virtual scenarios and objects through a magnetic tracking sensor generally placed over the hand (that permits a 3D position tracking of the end effector). Furthermore, cognitive tasks are generally coded as pick and place activities, ordering activities, selection activities, and sequential selection.

During the training, the patient was sitting in front of the device, actively interacting with the platform. The VRRS cognitive training was based on a dynamic interaction between the therapist and the patient, administering a virtual approach, using an augmented feedback with the a repetitive stimulation of specific domains such as orientation (personal, temporal, spatial orientation), memory and attention processes and verbal and not exsecutive functions, according to a task oriented use, choosing ad hoc software dedicate for each injured abilities.

The VRRS motor program includes about 40 specific tasks of the virtual sensory motor to stimulate muscle strengthening, strengthen leg tendons and ligaments, improve posture, pelvis movements, and balance reactions. All virtual exercises have been planned and organized by the therapist (after consultation with the neurologist), with increasing difficulty in relation to the time of execution and the category of activity. In the first training phase, the therapist carried out the preparation of the patient's muscle tissues by manipulation, before doing the motor activities, then the patient performed activities for trunk control and balance in the sitting position. To increase active movements in the upper limbs, manipulative exercises were proposed with the grip sensor module, which includes a mouse-like sensor that allows the patient unable to use the touch system to carry out the activities of gripping, selecting, pointing and reaching (see Fig. 1).

### 2.5 Outcomes

The patient well tolerated both the treatment protocols without any side effects or fatigue.

At the end of the traditional treatment (T1) using standard techniques, the patient presented a mild improvement only in head and trunk control as well as in depression symptoms. Only at the end of the combined approach with the VRRS, we observed a significant improvement in communicative abilities with a special reference to communication initiative, collaboration and motivation. Moreover, a substantial reduction in anxiety's level, depressive symptoms, apathy, indifference and disinhibition with the optimization of emotional attitude as per Hamilton Rating Scale for Depression and Brief Psychiatric Rating Scale score were noted (see Table 4).

**Table 4:** Cognitive outcome evaluated at baseline (T0), after the standard training (T1), at the beginning of the combined experimental approach (T2), and after such approach (T3).

		Conventional treatment			Advanced treatment VRRS-evo Cogn + PT		
Test	Domain	T0	T1	**RCI**	T2	T3	**RCI**
MMSE	Global Cognition	14.8	12.8		12.8	14.8	
CDT	Spatial dysfunction and neglect	5	4		4	5	
BPRS	Psychiatric Symptoms	64	58		60	52	**2.1**
FCS	Functional Communication	27	25		25	30	**2.2**
FAB	Executive Function (verbal and not)	10.9	8.9		7.9	10.9	
HRS-D	Depression Symptoms	14	20	**1.9**	19	11	**2.1**
DRS	Disability Category	17	16		17	17	
FIM	Functional Status	21	19		20	21	
TCT	Motor/Trunk impairment	24	22		22	26	**1.9**

BPRS (Brief Psychiatric Rating Scale) range 0–126; CDT (Clock Drawing Test) range 0–10; cut off = 6; DRS (Disability Rating Scale) range 0–29; FAB (Functional Assessment Battery) range 0–18; cut off = 12; FCS (Functional Communication Scale) range 0–22; FIM (Functional Independence Measure) motor (items 1–13): 13–91; cognitive (items 14–18): 5–35; HRS-D (Hamilton Rating Scale for Depression) range 0–24; cut off = 7; MMSE (Mini Mental State Examination) range 0–30; cut off = 24; total (items 1–18): 18–126TCT (Trunk Control Test) range 0–100.

## 3 Discussion

To the best of our knowledge, this is the first report on effective advanced training in the treatment of both motor and cognitive deficit in a patient with NHD.

This rare autosomal recessive inherited disease is caused by the mutation of the TREM-2 gene, which is involved in the complex signaling networks of osteoclasts and microglia.^[33]^ Then, NHD is characterized by progressive dementia and bone cysts with skeletal x-ray radiolucency. Most patients already at a young age experience bone symptoms, with pain in the hands, feet and knees as well as an increased susceptibility to pathological fractures. Dementia is mostly characterized by personality changes, and patients experience euphoria, indifference, slouchy lifestyle, apathy, disinhibition, and a lack of understanding of their illness.

Nowadays the diagnosis of NHD is made, according to the clinical picture, skeletal x-ray showing cystic and radiolucent lesions in long bones, and brain imaging, which shows findings of ventricular enlargement and atrophy of the cerebral hemisphere, predominantly in the frontal and temporal lobes.^[34]^

Unfortunately, once diagnosis is made, no specific drug or therapeutic approach exists, and this is why there is a need to pave the way for a better management of this rare disease. Our case study shows the importance of using advanced neurorehabilitation in an attempt to slow down the unstoppable course of NHD, reduce psychiatric symptoms, and maintain motivation as well as motor and cognitive outcomes for as long as possible. A “multimodal” approach is therefore more useful to better manage the complex psychological, cognitive and motor aspects of the disease. In fact, as current scientific literature does not give any type of therapeutic-rehabilitative indication or guidelines for the treatment of NHD patients, this single case-study may be the basis for implementing a strategic and innovative, and potentially effective, rehabilitative approach.

The idea of using this novel training stems from the growing evidence that VR could be effective in different patient populations, including those affected by neurological (such as stroke, traumatic brain injury and multiple sclerosis), psychiatric and other motor disorders.^[35–43]^ According to this data, we found that VR led to better results than conventional training, as in our patient it promoted a reduction of depressive and psychiatric symptoms, an improvement in motor-cognitive function and communication abilities with a higher engagement and participation thanks to dynamic gaming interaction. Indeed, its gaming-like structure activates a key motivational mechanism for the achievement of rehabilitation goals. Notably, it has also been demonstrated that VR technology, providing a higher feedback on the characteristics of motion, can improve motor learning and performance in both healthy and post-stroke subjects compared to traditional training.^[44]^

Among the several existing tools, the VRRS allows the creation of different simulated scenarios so that the therapist can prepare a patient tailored rehabilitation. Indeed, it allows the calibration of the difficulty of proposed activities based on the actual capabilities and the potential of the patient which the therapist is dealing with. In addition, it is possible to constantly measure and monitor performance by providing a wide range of responses. Indeed, VRRS is designed to put the patient in a situation that generates increased feedback to his/her CNS (augmented feedback) through exercises performed in a virtual environment helping to develop the knowledge of the results of movements (knowledge of the results) and the knowledge of the quality of movements (knowledge of performance), leading to a training-specific motor learning.^[45,46]^ In this way, the CNS can activate a physiological learning mechanism called “reinforcement learning” which implies an increase in the specific information of a movement to produce an effective improvement in performance quality.^[47]^ This boosting in neural plasticity with a consequent potentiation in the functional outcomes could be why our patients achieved higher results after this novel approach than following the conventional training.

We are aware that findings from a single case report have many limitations, including epidemiological bias, impossibility of causal inference and generalization and over-interpretation. Thus, our results should be confirmed by well-designed clinical trials, taking into account long-term effects of this novel training.

## 4 Conclusion

With this single case report, we want to underline the important role of advanced neurorehabilitation approaches using VR in potentiating and maintaining functional outcomes in patients with neurological disorders, including those affected by rare dementias. In fact, after the innovative training our patients affected by NHD achieved better results regarding both motor and cognitive function. Future larger sample studies are needed to confirm these promising findings and to investigate whether and to which extent the outcomes persist over time.

## Author contributions

**Conceptualization:** Rosaria De Luca and Rocco Salvatore Calabrò.

**Data curation:** Mirjam Bonanno, William Torregrossa, Marella Buda, and Maria Mucari, Angelo Caminiti.

**Investigation:** Carmela Rifici, Patrizia Pollicino, Angela Marra, and Angelo Caminiti.

**Methodology:** Mirjam Bonanno, William Torregrossa, Marella Buda, Maria Mucari, and Angelo Caminiti, Angela Marra.

**Supervision:** Rosaria De Luca and Rocco Salvatore Calabrò.

**Validation:** Rosaria De Luca and Rocco Salvatore Calabrò.

**Visualization:** Angela Marra.

**Visualization:** Carmela Rifici, Patrizia Pollicino, and Angela Marra.

**Writing – original draft:** Rosaria De Luca, Mirjam Bonanno, and William Torregrossa.

**Writing – review & editing:** Rocco Salvatore Calabrò.
